# Generation of a *Litopenaeus vannamei* hepatopancreas cell atlas from single nuclei transcriptomics using a new nuclei isolation method

**DOI:** 10.1186/s12864-025-12475-z

**Published:** 2025-12-28

**Authors:** Alexandra Florea, Rose Ruiz Daniels, Sarah J. Salisbury, James Furniss, Diego Robledo, Tim P. Bean

**Affiliations:** 1 The Roslin Institute, University of Edinburgh, Edinburgh, EH25 9RG United Kingdom; 2 University of Stirling, Stirling, FK9 4LA United Kingdom; 3 University of Exeter, Exeter, EX4 4PY United Kingdom; 4 University of Santiago de Compostela, Santiago de Compostela, 15705 Spain

**Keywords:** Penaeid shrimp, *Litopenaeus vannamei*, Transcriptomics, Single nuclei RNA sequencing, Hepatopancreas, Cell atlas

## Abstract

**Background:**

Crustacean aquaculture is one of the most important food sectors globally and projected to grow. It is a source of nutritious and economic animal protein in many countries. As the global demand for sea food increases, and with an increase in climatic and pathogenic threats to the industry, curating our current knowledge about crustaceans, as well as generating new tools and resources to help minimise the impact of various diseases on the sustainability of the industry is of the utmost importance to increase the resilience of the farmed animal stocks. The main aim of this pilot study was to create a new cell atlas for Pacific whiteleg shrimp (*Litopenaeus vannamei*) hepatopancreas which encapsulates both the different hepatocyte cell states as well as the various supporting cells found throughout the hepatopancreas, while developing a new method for nuclei isolation and data analysis in the species.

**Results:**

We developed new protocols for TST-based nuclei isolation which could be used to successfully isolate and process crustacean-derived nuclei for single nuclei RNA-sequencing analysis with minimal nuclei degradation using frozen hepatopancreas tissue from healthy *L. vannamei* adults. The bioinformatic analysis that followed allowed us to create a new cell atlas for the hepatopancreas which details the different hepatocyte cell states. Additionally, we built up on the existing knowledge by also analysing the multiple supporting cell clusters such as IECs, fibroblasts and myocytes, which helps improve our understanding of the characteristics of this immune-related organ.

**Conclusion:**

Overall, 4005 cells were assigned to nine different clusters. Distinct marker genes suggest unique functions of each hepatocyte subtypes and adjacent supportive cells. The new TST-based isolation method for frozen or archived nuclei is particularly useful for processing difficult samples, such as the hepatopancreas, minimise stress and dissociation bias while allowing for greater flexibility between tissue sampling and processing times.

Combining the knowledge gained through this study with past and future work in other penaeid shrimp species will allow us to create a powerful resource that will help uncover new knowledge about these important species, especially in the field of stress and immunity.

**Graphical Abstract:**

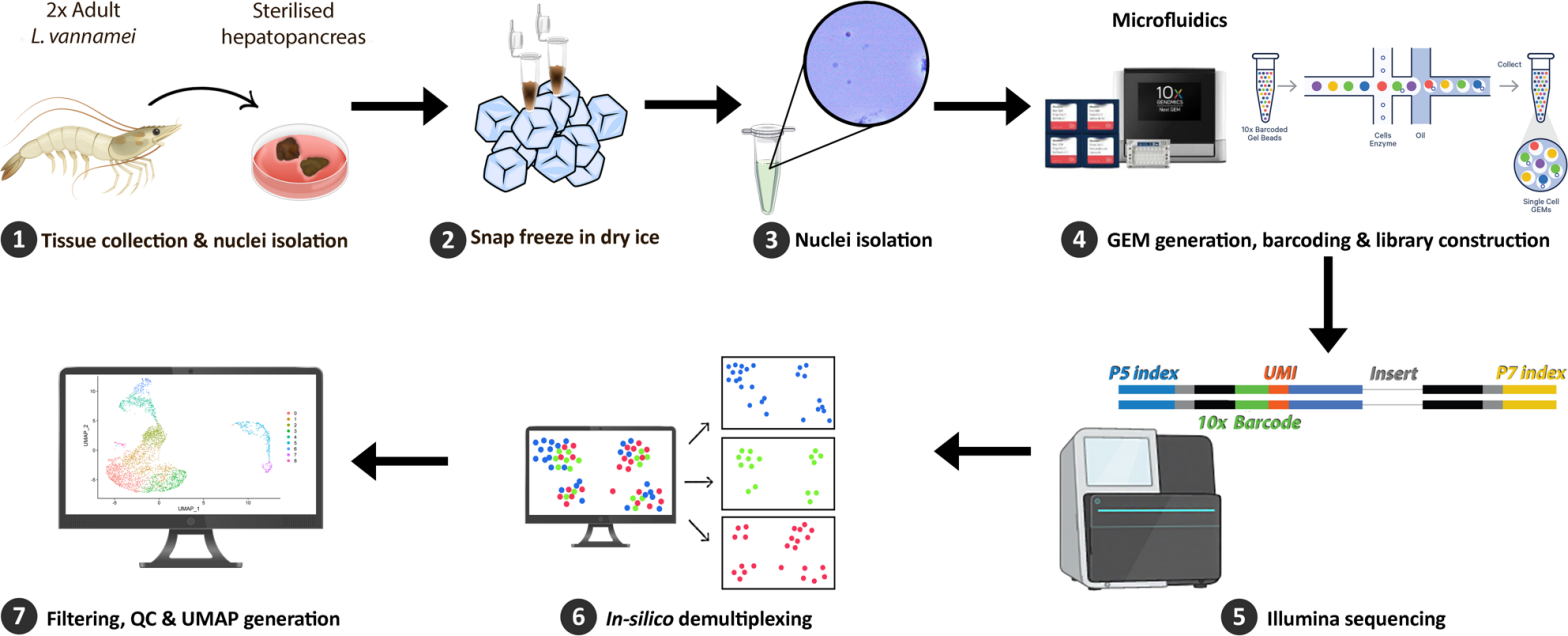

## Background

Penaeid prawns, such as Pacific whiteleg shrimp (*Litopenaeus vannamei*), Chinese white shrimp (*Fenneropenaeus chinensis*), kuruma shrimp (*Marsupenaeus japonicus*) and black tiger shrimp (*Panaeus monodon*), accounted for 62.2% of all farmed crustacean species, and remain the second most valuable farmed group after salmonids, accounting for 17% of total global aquaculture value [1]. Whiteleg shrimp was the top produced aquaculture species globally, at 6.8 million tonnes.

Despite their global productivity, shrimp farms frequently face disease outbreaks which severely hamper production [2]. Unlike mammals and other higher vertebrates, penaeid shrimp lack antibody-mediated adaptive immunity, relying solely on effective cellular and humoral innate immunity [3, 4]. While the immune mechanisms in penaeid species has been studied for years, there is still certain ambiguity about the exact roles certain immune organs have, the cell types that compose them, and the transcriptomic profiling of these cell types.

Together with the lymphoid organ and haemocytes, the hepatopancreas plays a crucial role in controlling the immune responses in penaeid shrimp. The hepatopancreas is a large, paired organ located in the cephalothorax of crustaceans, flanking the stomach and anterior midgut [5]. It is analogous to the vertebrate liver and serves as the major detoxification organ and largest digestive organ responsible for nutrient absorption, metabolism and enzyme synthesis. It largely combines several of the functions of the liver, pancreas, and intestine of vertebrates [6–8]. It also has an important role in haematopoiesis and immunity by producing many defence proteins, including hemocyanin, which is a precursor for antimicrobial peptides, and lectin, a protein involved in immune response against pathogens [9, 10]. Santos et al., 2020, have indicated that there are many genes upregulated in the hepatopancreas of healthy shrimp which are among the most important ones when it comes to keeping shrimp pathogen-free, like in the case of white spot syndrome virus (WSSV) [9]. Unfortunately, many of the highly important immunological proteins as well as their mechanisms of action continue to elude scientists [9]. New studies that look at the genetics, genomics and physiology of prawns are necessary to complete this immune puzzle, accompanied by an ever-increasing need for better genome sequences and annotations.

Structurally, there are four main types of hepatocytes found in crustacean hepatopancreas tubules: resorptive/absorptive cells (R-cells), blister-like cells (B-cells), fibrillar cells (F-cells), and embryonic cells (E-cells) [10]. E-cells, located at the distal tips of each tubule and characterized by proximal nuclei and prominent nuclear bodies, serve as progenitors for the other three cell types within the digestive gland of crustaceans [10, 11]. R-cells, also known as Restzellen, are multi-vacuolated cells dispersed throughout the hepatopancreas that play a crucial role in the absorption and storage of lipids and glycogen. Additionally, they sequester various mineral deposits, including calcium, magnesium, phosphorus, and sulphur, essential for a variety of metabolic processes [10, 11]. B-cells, or Blastozellen, are large primary secretory cells that are the main producers of digestive enzymes which facilitate the breakdown of food particles, allowing for nutrient absorption. They also contribute to nutrient accumulation, intracellular digestion, and the transport of digested material to other parts of the organ for further processing [10, 11]. Lastly, F-cells are responsible for protein synthesis and the storage of minerals. These cells are integral to maintaining the structural integrity of the hepatopancreas and ensuring the proper synthesis of proteins necessary for various cellular functions and metabolic activities [10–12]. F-cells are also responsible for producing hemocyanin, a protein of high importance in shrimp immunity [10]. In addition to these specialized cells, the hepatopancreatic tubules also contain musculature which aids in the movement of the digesta through the organ [13]. The hepatopancreatic tubule is epithelial in nature and is wrapped in a connective tissue capsule [14].

Although the hepatopancreas has been well-studied histologically and morphologically, the transcriptomic signatures of the cell types present within this organ remain largely unknown. The hepatopancreas is known to play a key role in immune and stress response, particularly via the F-cells which function in synthesizing the hemocyanin monomers and pathogen recognition molecules [11], however, very little is known about the underlying transcriptomic profiling that contribute to this immunity.

In order to elucidate the underlying genetic mechanisms of prawn immunity, we first need to have a good foundation of their “regular” state. Meaning that we need to understand the individual components of immune-related organs as well as their specific gene expression. This will help us build on this knowledge in the future using more complex stress and pathogen challenges. In this study we therefore generated a cell atlas using single nuclei RNA sequencing (snRNA-seq) to transcriptomically profile the cell types present within this critical organ. Our study aimed to identify the different cell types found in hepatopancreas including both functional and structural-type cells, with particular interest to immune-tissue cell populations and sub-populations.

## Methods

### Experimental animal care

Adult *L. vannamei* weighing 6–12 g were sourced and kept at 28 ± 0.5 °C, in aerated artificial seawater with a salinity of 25‰ and pH of ~ 8.0. A 12-hour daylight cycle was upkept throughout the entire housing period. The shrimp were fed a commercial shrimp pellet diet twice a day (~ 5% body weight/day). Solid waste syphoning and water tests (pH, ammonia, nitrite, nitrate, dissolved oxygen and salinity) were performed daily. Water exchanges (1/3 of aquarium volume) were performed every other day.

### Sampling and nuclei processing

#### Tissue sampling

**Fig. 1:**
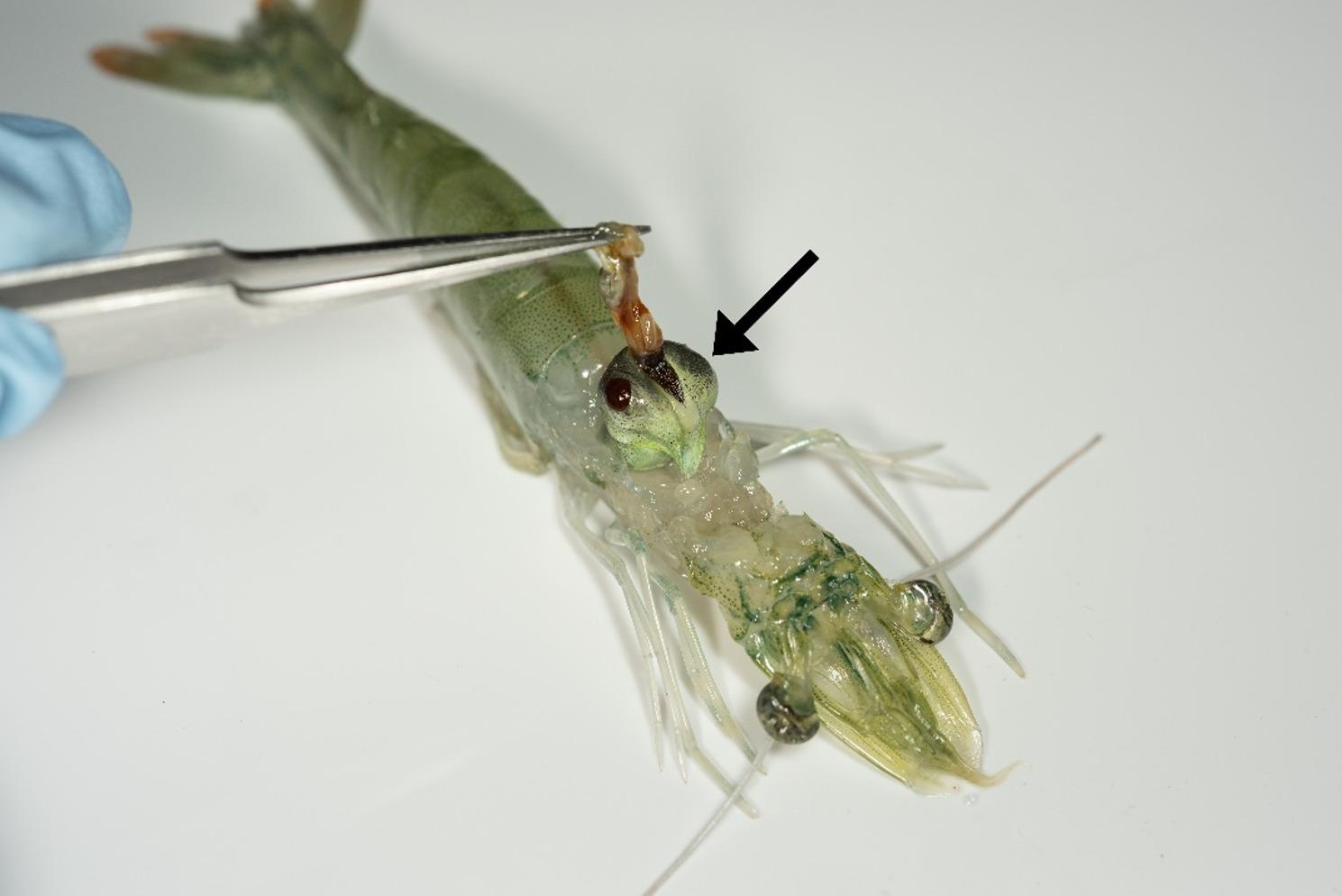
Pacific whiteleg prawn dissection. Dissection of hepatopancreas prior to flash freezing for single nuclei transcriptomic analysis via single nuclei RNA sequencing. The black arrow indicates the hepatopancreas

Several adult *L. vannamei* shrimp were randomly samples from a pool of healthy shrimp and sacrificed by immersion in an ice-slurry bath for a minimum of 10 min. The shrimp were confirmed dead once no heart or gill movements could be seen through the carapace. The shrimp were then checked for experimental suitability, i.e., 7–10 g body weight, moult stage C (inter-moult) and healthy-looking hepatopancreas. The hepatopancreas was dissected aseptically (Fig. 1) and then snap-frozen on dry ice. The tissue samples were stored at -80 ° C until processing.

#### Nuclei isolation and fixation

Nuclei were extracted using a modified version of the TST-based method [15]. Briefly, we prepared the 2X ST buffer (stock of salt-Tris solution; 146 mM NaCl, 10 mM Tris-HCl pH 7.5, 1 mM CaCl2, 21 mM MgCL2), 1X ST-buffer solution working solution (Dilute 2x ST 1:1 in nuclease-free water with addition of RNAse Inhibitor to final conc. 40 U/mL) and TST working solution, enough for two samples (2 mL of 2X ST buffer, 20 µl BSA 2% and 1.86 mL of dH_2_0). Note that the hepatopancreas nuclei were extracted without the addition of Tween-20 (as in Ruiz Daniels et al.., 2023 [15]) due to the nuclei already being released during the freeze-thaw process in TST buffer, meaning further use of Tween-20 would have led to degraded nuclei. Extractions using Tween-20 have been tried in prior experiments, which led to poor isolations and highly degraded nuclei, unsuitable for further processing.

For each sample, a 6-well tissue culture plate was placed on ice and 1 mL of TST buffer was added to a well. The frozen tissue sample (10–50 mg) was placed in the buffer straight from dry ice. The samples were macerated as finely as possible with spring scissors for one minute. The sample was then sequentially pipetted for 30 s with a P1000 and 30 s with a P200. The lysate was then filtered through a 40 μm Falcon cell-strainer into a clean well of the plate, and then again through a 20 μm cell strainer. This was done in order to remove the majority of the debris found in the sample that would otherwise interfere with the sample processing in the Chromium machine (i.e. block the capillaries and prevent the formation of the emulsion). 1 mL of TST was added to wash the initial maceration well and the liquid was passed through the filters again to ensure that no nuclei remained on the filter. The volume was brought up to 5 mL with the addition of 3 mL of 1X ST buffer. The lysate was transferred to a 15 mL Falcon tube and centrifuged at 4 °C for 5 min at 500 g. The pellet was resuspended in 20 µL of 1X ST (with RNAse inhibitor to a final conc. 40 U/mL) for each 1 mg of starting tissue (i.e., 200 µL for 10 mg, 1 mL for 50 mg).

### RNA library generation and sequencing

Nuclei subsamples were stained with trypan blue and visually inspected to determine nuclei quality and manually counted using a Neubauer Haemocytometer. For each sample 7,000–7,500 nuclei were loaded onto a Chromium Chip. The samples were partitioned and barcoded at the single cell level using the microfluidic system Chromium Controller from 10X Genomics. The resulting emulsion was processed using the Chromium Next GEM Single Cell 3ʹ Reagent Kits v3.1 (Dual Index) from 10X Genomics according to manufacturer’s instructions. The resulting libraries were sent to Genewiz (Azenta) for sequencing using Illumina (2 × 150 bp, 350 M, dual index). The sequencing was done using 5% PhiX and approximately 30,000 reads/cell (overall, 400 million reads across 2 samples).

The full sequencing dataset has been uploaded to the SRA database, under BioProject accession PRJNA1302321. The two 10x Libraries can be found under BioSample accessions: SAMN50468451 and SAMN50468452.

### Data analysis

The data analysis was performed using a homemade pipeline (available: https://github.com/Roslin-Aquaculture/10x-snRNAseq_LV-HP-atlas) that combined STARsolo ver. 2.7.10a for Linux and Seurat single cell analysis package for R (v4.2.0) [16] for R. Below is an overview of the analysis steps undertaken. The full details, including individual sample thresholds for filtering can be found inside the GitHub repository.

Firstly, the data was demultiplexed into biological samples in STARsolo ver. 2.7.10a [17], using the *Litopenaeus vannamei* (Genome assembly ASM378908v1, NCBI RefSeq assembly GCF_003789085.1) annotation. Reads were aligned against the genome and annotation, and gene expression per cell estimated, resulting in a unique molecular identifier (UMI) count matrix per sample. Low-quality sequences were removed at this stage.

The demultiplexed data were pre-processed prior to Clustering and Differential Gene Expression (DEG) analysis, including QC, was performed in Seurat (v4.4.0) [18] (SeuratObject v 4.0.4). The Seurat object was created after removing nuclei with fewer than 200 features and features occurring in fewer than three nuclei. Upper and lower thresholds for UMI and feature counts per nucleus were then determined for each sample based on knee plot visualization. For all samples, only nuclei with more than 750 UMIs but less than 3500 UMIs and more than 300 features but less than 1500 features were retained. Cells which contained more than 5% mitochondrial DNA genes (features) were removed. This was followed by the SCT and cell cycle data normalisation steps.

Linear dimension reduction was conducted using the “RunPCA” function with 50 PCs. After consulting the elbow plot, a UMAP using 10 PCs was run, and the “FindNeighbours” function was applied using 10 PCs before using the “FindClusters” function with a resolution of 0.4. The data was normalised a second time using an SCT assay after which DoubletFinder package was used to remove the doublets from the dataset by selecting the pK values with the highest associated BCmvn values.

A final UMAP of the tissue was then generated (10 PCs and a resolution = 0.4 for clustering). Differential gene expression analysis using a Logistic Regression Model was performed (logfc.threshold = 0.25) in order to determine the gene markers for each cluster. The differentially expressed genes were calculated using the Seurat FindAllMarkers function, which applies the Wilcoxon rank sum test with default cut-offs (multi-test adjusted p-value < 0.01, log2-fold change > 0.25, with expression of the gene in at least 25% of nuclei in the cluster tested).

At this point, a list containing the top 20 marker genes for each cluster was generated. Each gene’s symbol, name, cellular and molecular function was identified manually with a combination of NCBI (www.ncbi.nlm.nih.gov/gene/), UniProt (www.uniprot.org/) and The Human Protein Atlas (www.proteinatlas.org/) via homology with human and (*Homo sapiens*) and rat (*Rattus norvegicus*) genes. Following this, the UMAP clusters were annotated, and a series of plots (Heatmaps, Dotplots, Feature plots, Violin plots) was generated characterising each cluster and its relevant markers.

## Results

### Nuclei isolation

**Fig. 2:**
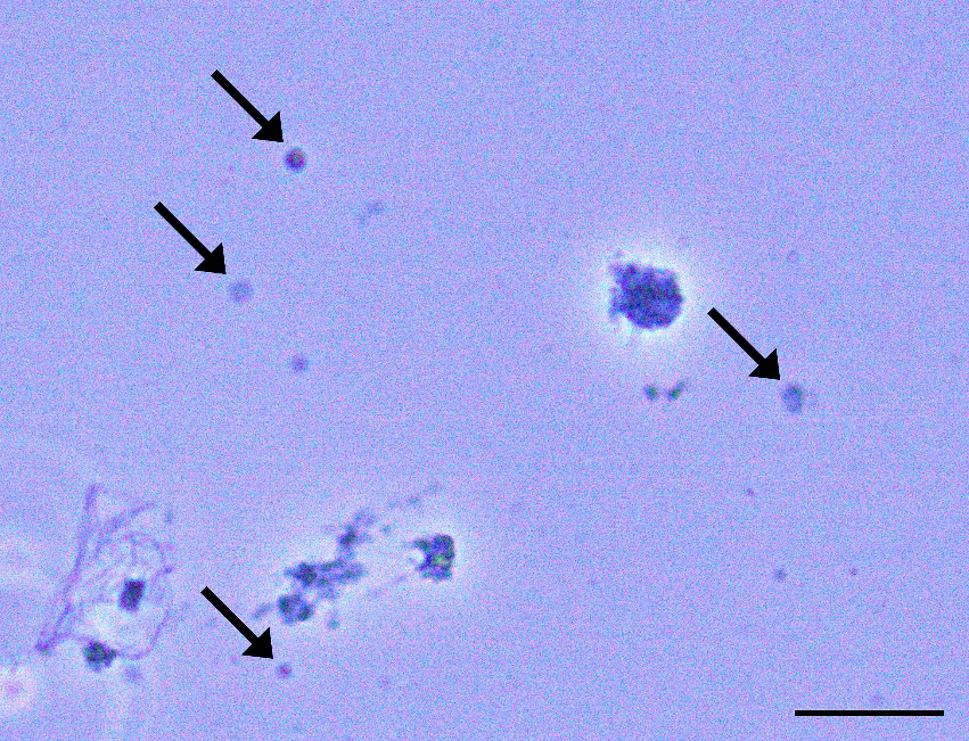
Nuclei isolated from hepatopancreas. Microscope picture showing Pacific whiteleg prawn hepatopancreas nuclei following TST-based tissue dissociation. The sample was stained with trypan blue for visual contrast. The black arrows indicate the isolated nuclei. The black scale line indicates 50 μm

Using the TST-based method, we isolated between 7000 and 7500 nuclei per sample. The resulting nuclei which, can be seen in Fig. 2, measure between 5 and 10 μm in size and do not display signs of degradation (smooth, round surface) which indicates that the TST-based method is effective in releasing the nuclei from the cells while also being gentle on the nuclear membrane.

Moderate debris could be seen in the nuclei samples even after filtering through both a 40 μm and a 20 μm cell sieve. The filtering was, however, sufficient to enable us to run the samples through the Chromium, resulting in both samples being processed to the emulsion state without any technical issues.

### Cell atlas construction and histopathological data integration

**Fig. 3:**
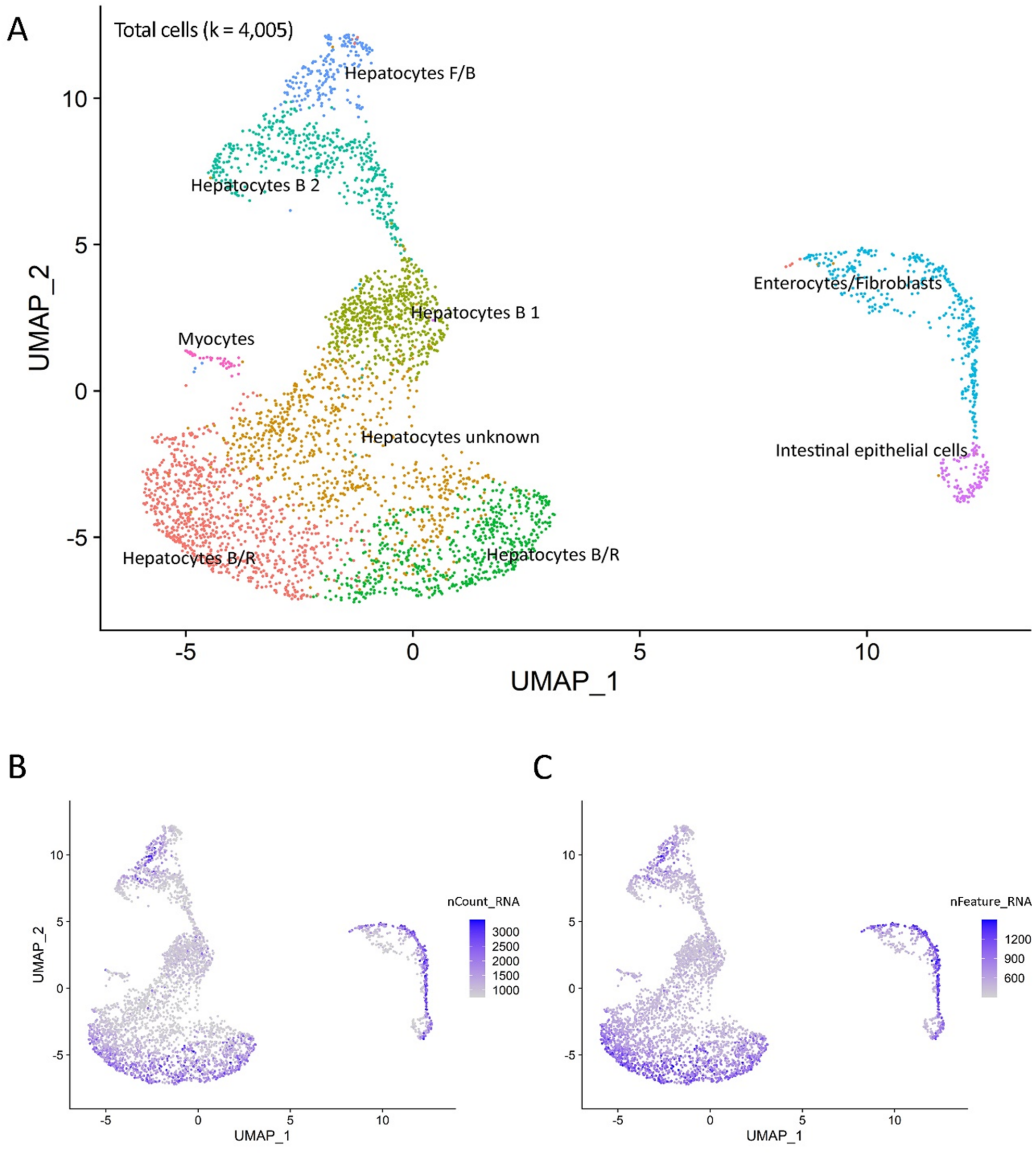
Hepatopancreas UMAPs of the cell types grouped by transcriptomic identity. (**A**) UMAP plot of cell clusters from shrimp hepatopancreas tissue. The different clusters (cell identities) are shown indifferent colours. (**B**) Total number of molecules (UMIs) detected within cells (nCount_RNA). (**C**) Total number of genes detected in each cell (nFeature_RNA)

**Fig. 4:**
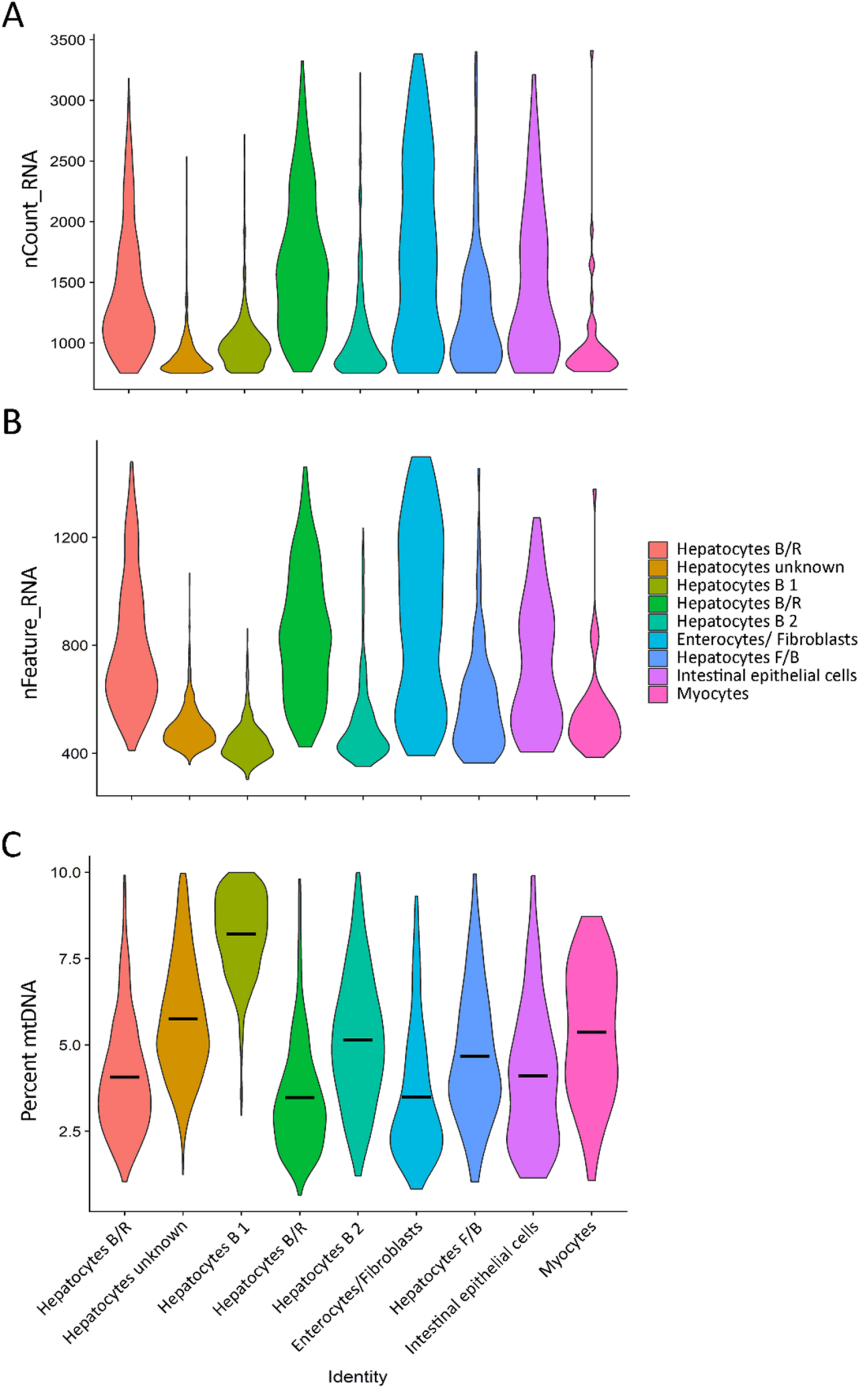
Hepatopancreas data quality violin plots by cluster. (**A**) Average number of molecules (UMIs) detected within cells by cluster (nCount_RNA). (**B**) Average number of genes detected in each cell by cluster (nFeature_RNA). (**C**) Mitochondrial DNA percentage by cluster

The initial filtering has identified one of our hepatopancreas libraries as unusable, due to the low number of nuclei that passed QC. The remaining sample was carried forward for downstream analysis. The STARsolo output showed that the sample had an initial 383 million reads (0.72 sequencing saturation), with an average 616 UMIs (Figs. 3B and 4A) and 294 genes per cell (Figs. 3C and 4B).

A total of 9 clusters were identified (Fig. 3A). The majority of clusters had distinct and recognisable transcriptomic profiles, with only one hepatocyte cluster (Cluster 1 – “Hepatocytes unknown”) having an undetermined profile due to the high number of uncharacterised marker genes and lack of cell type specificity of the annotated marker genes. The identification of the distinct hepatopancreatic clusters was made possible due to the histopathological data provided by WOAH Collaborating Centre for Emerging Aquatic Animal Diseases. This dataset described the four distinct hepatopancreas cell types (i.e., Fibrillar F-cells, Reserve/Resorptive R-cells, Blister B-cells and Embryonal/stem E-cells), as well as their cellular roles, making it easier to assign cluster identities based on the gene expression profile.

### Cell cluster identity

**Fig. 5:**
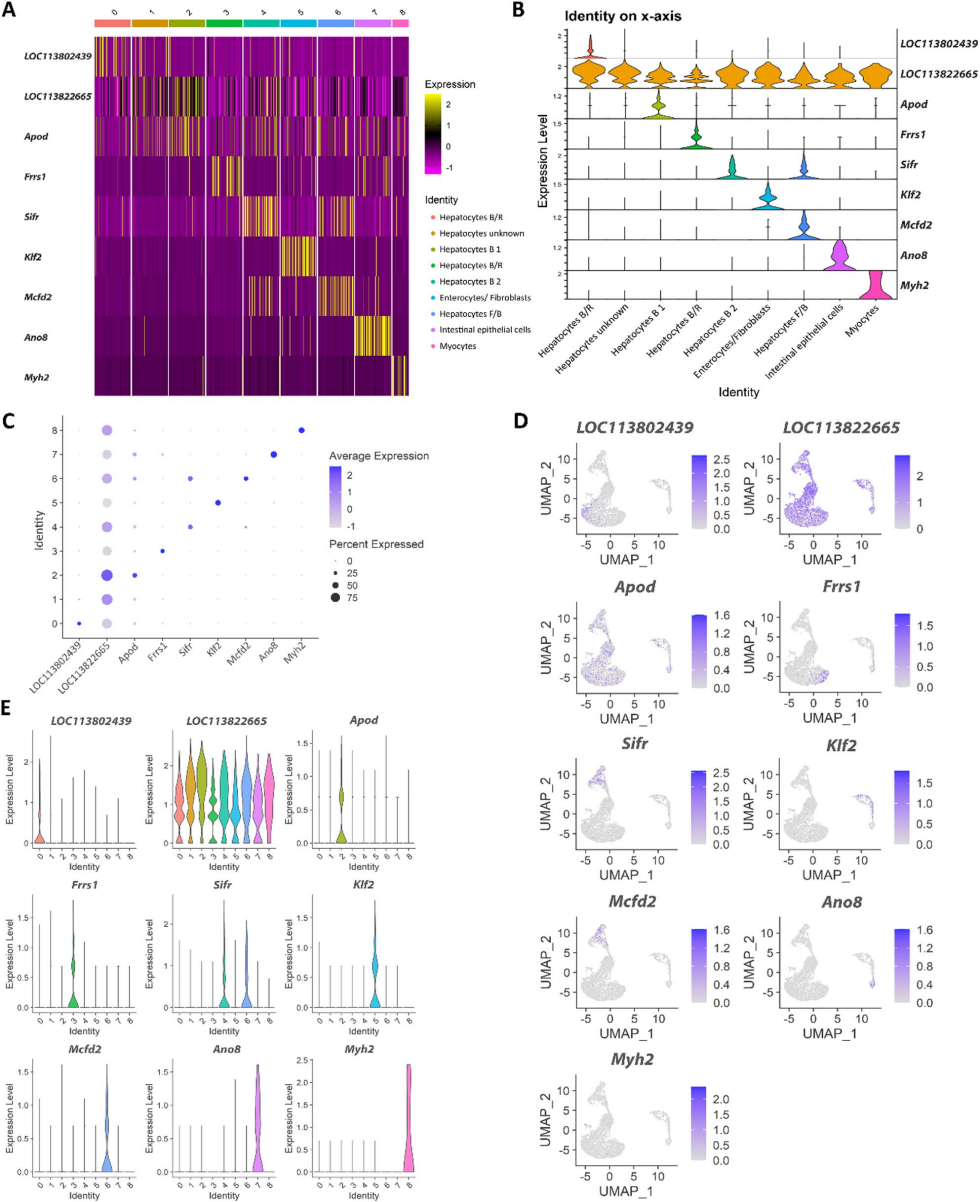
Hepatopancreas cluster analysis. (**A**) Heatmap of the cluster marker genes in hepatopancreas. (**B**) Violin plots of the significant marker genes per cluster. The top marker gene for each cluster is specified on the right side. (**C**) Dot plot of significantly marker genes per cluster showed as an Average Scaled Expression. (**D**) UMAPs showing the expression level and location of the top cluster markers. (**E**) Violin plots showing the expression of the marker gene for each cluster

Figures 3A and 4, and Fig. 5 detail the cluster profile of the hepatopancreas cell atlas derived from healthy adult shrimp, which include both hepatocyte-specific cell clusters as well as other functionally-distinct cells.

#### Hepatocyte cell clusters

Cluster 0 was assigned to “Hepatocytes type B/R” due to the mix of expressed genes that have a role in digestion (*Ace*, and two paralogues of *Atp1a1*) and tubular reabsorption (*Slc6a18*,* Bbox1*). Cluster 1 remains as an unknown type of hepatocyte as many of its marker genes were not exclusive to this cluster, while its unique marker is an uncharacterised locus. Cluster 2, “Hepatocytes type B 1” expresses several marker genes with roles in digestion or macromolecule degradation (*Cpb1*,* Amy1a*,* Peritrophin*,* Ctrb1*,* Fuca1*). Cluster 1 and Cluster 2 have a higher average mitochondrial percentage (Fig. 4C) as well as a lower average UMI and gene count per cell than other clusters (Fig. 4A, B), which could indicate a higher fraction of apoptotic or dying cells (possibly due to the presence of digestive enzymes in and around these cells). Cluster 3, “Hepatocyte type B/R”, expresses genes related to lipid (*Capn5*) and glucose (*Q8ted4*,* Crhr2*) metabolism, although confidence in this cluster identity is lower than the other known hepatocyte clusters due to many of the cluster’s top features being unannotated. Cluster 4, “Hepatocytes type B 2”, has multiple markers with roles in pancreatic digestion (*Cpa1* and two *Prss1* paralogues), enteroendocrine and proximal tubular cells digestion-related genes (*Vwa7*) as well as multiple genes with roles in liver and kidney transport and metabolism (*Acsm3*,* Arsb*,* Slc7a8*,* Cyp2l1*). Cluster 6, “Hepatocytes type F/B”, identity is derived from a mix of digestion-linked (*Vwa7*, two *Prss1* paralogues) and secretion-linked genes (*Amy1a*,* Mcfd2*).

#### Non-hepatocyte cell clusters

Cluster 5 has markers with both enterocyte-like (six *Malrd1* paralogues), and fibroblast-like profile (*Csgalnact-1*,* Myb*), thus placing it as an “Enterocytes/ Fibroblasts” cluster. Cluster 7 is identified as “Intestinal epithelial cells”, with genes that have a role in absorption (*AnxB9*,* Picot*) and indicate a profile similar to absorptive epithelial cells. The last cluster, the “Myocytes”, has a very clear profile, with multiple genes coding for proteins involved in muscle contraction (*Myh2*,* Tbc5*,* Unc-22*,* Tnni3* and two *Ttn* paralogues).

## Discussion

Transcriptomic studies via RNA sequencing are becoming a staple technique in disease and stress-focused studies in Pacific whiteleg shrimp. The hepatopancreas, in particular, has been the focus of many such RNA sequencing studies over the years, covering a wide range of stressors, from ammonia [19], lead [20] and eye ablation [21]; to pathogens such as vibrio [22, 23], *Enterocytozoon hepatopenaei* parasite [24] and Taura Syndrome Virus [25].

While RNA sequencing is providing great insight into the genes and pathways involved in different stress and infection mechanisms, the development of routine single cell and single nuclei transcriptomics is allowing us to have a closer look at cell-by-cell responses and into the individual roles of different cell types within a tissue or organism. The last five years have seen a “bloom” of single cell RNA-sequencing studies in penaeid prawn species, focused on haemocytes [4, 24, 26–29] and hepatopancreas [10, 27].

The current transcriptomic study was performed via single nuclei RNA sequencing on adult *L. vannamei* hepatopancreas, a crucial component of the immune system of the shrimp. This is the first study that uses single nuclei technology on penaeid shrimp hepatopancreatic tissue, rather than the widely used single cell method. During our study, we successfully developed a new protocol for nuclei isolation and processing from shrimp samples which allowed us to extract intact nuclei from frozen tissues (Fig. 2) and process them using 10X Genomics technology. This protocol can be used in future studies which require the use of frozen or archived samples or those that can benefit from a flexible timeframe between sample collection and processing.

**Fig. 6:**
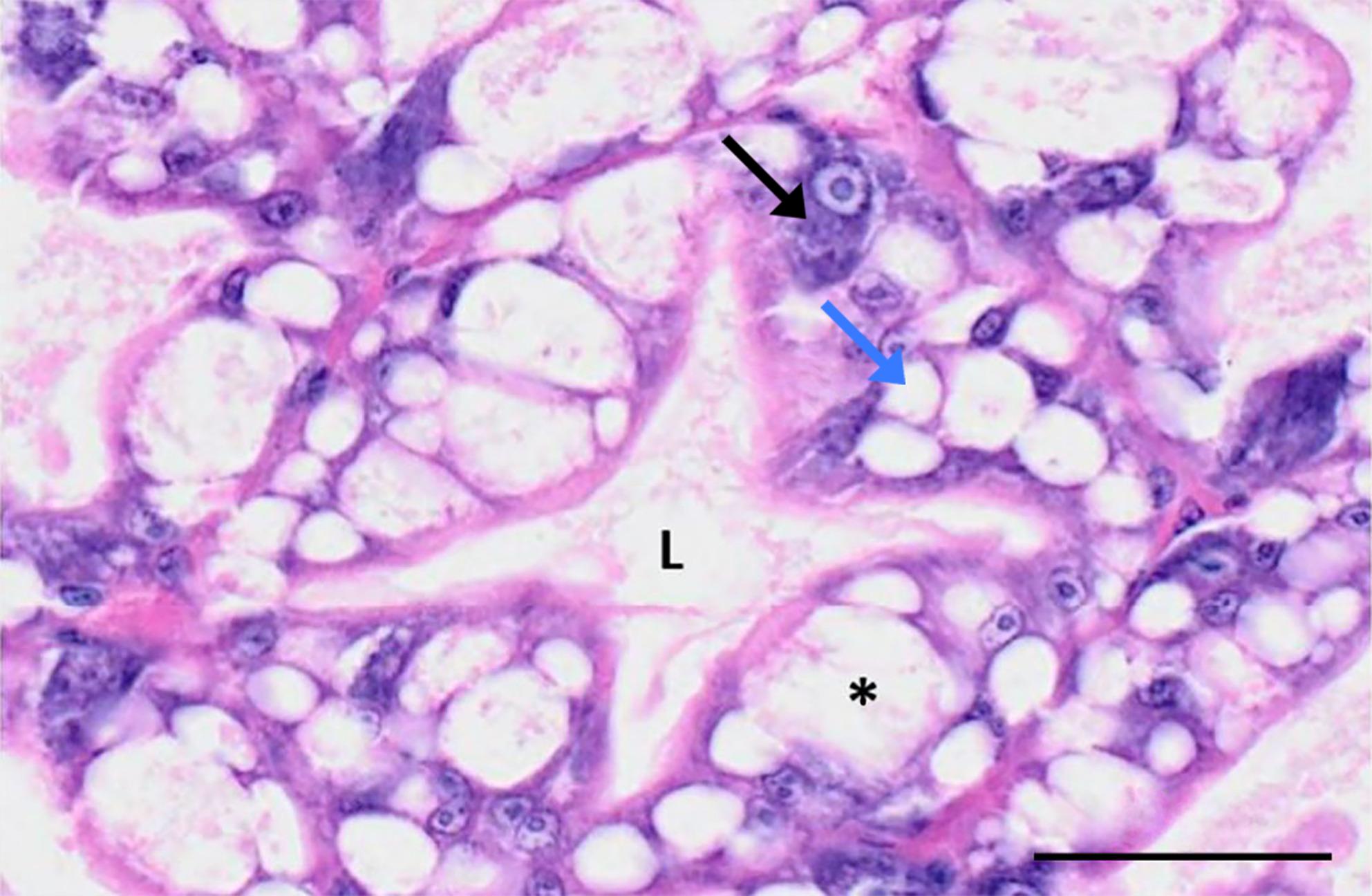
Histopathology of penaeid shrimp hepatopancreas. Prawn hepatopancreas tubule displaying Fibrillar F-cells (black arrow), Reserve/Resorptive R-cells (blue arrow) and Blister B-cells (asterisk). Annotated image provided by WOAH Collaborating Centre for Emerging Aquatic Animal Diseases

The resulting sequencing data allowed us to create a detailed cell atlas and identify multiple cell types with distinct roles in tissue biology. This atlas includes not only hepatocyte cell states, but also different cell types with supporting roles which help round up the transcriptomic knowledge of this important immune organ. There are four main types of hepatocytes found in crustacean hepatopancreas tubules: R-cells, B-cells, F-cells, and E-cells [10] (Fig. 6). R-, B-, and F-cell types have been successfully identified in the study, although the exact function of the cluster has been elusive at times due to the gene expression profile (Clusters 0, 3 and 6) (Fig. 3A). The last hepatocyte subtype, the embryonic cells (E-cells), which are located at the distal tips of each tubule and serve as progenitors for the other three cell types within the digestive gland of crustaceans, remained elusive. Zhu et al. (2022) have identified E-cells using key genes in the “hedgehog signalling” pathway, such as Ccnd2 [10]. While we could not find distinctive markers for E-cells, it is possible that Cluster 1 contains E-cells or somewhat undifferentiated cells due to the variety of cluster gene markers, some of which were common to the other cell clusters. This pattern of cluster markers (i.e. high expression of multiple marker genes) was also previously found by Li et al.., 2022. Their Hep1 cluster was speculated to be a developmental starting point for hepatocytes, with their findings backed up by a pseudotime analysis that looked at the developmental directions of the different clusters [27].

The 2022 study by Zhu et al.. that looked into cold tolerance DEGs in *L. vannamei* using hepatopancreas samples has also identified a similar hepatocyte cluster structure (with four distinct hepatocyte types) and gene expression as the one seen in this study, although no further functional or supportive cell types were found [10]. Our analysis revealed many more clusters including supplementary cell types but also a separate group of intestinal epithelial cells suggesting greater cellular complexity to this organ. It is worth noting that the present study did not find as clear-cut differences among hepatocytes as in Zhu et al.., (2022), which could potentially reflect some lability between the different hepatocyte types [10].

In addition to the specialized cells, the hepatopancreatic tubules also contain musculature of epithelial nature which aids in the movement of the digesta through the organ [13]. These types of cells were successfully identified (Cluster 8). The hepatopancreas is also wrapped in a connective tissue capsule [14] which was identified as Cluster 5. Other epithelial cells, which form protective layers and linings on surfaces throughout the body and play roles in absorption, secretion, and sensation have also been identified (Cluster 7).

The present study can be used as foundational knowledge for future experiments looking at environmental stressors, similar to the work done by Li et al., 2022 where they looked the transcriptomic responses of haemocytes and hepatopancreas under ammonia stress [27]; Zhu et al.., 2022 where they investigated cold tolerance gene expression profiles in hepatopancreas [10]; and Lian et al.., 2023, which looked at toxicity mechanisms in shrimp haemocytes subjected to nitrite stress [30]; as well as a baseline transcriptomic profile and cell composition for future pathogen challenge studies, such as those done by Cui et al., 2024 and Koiwai et al., 2023 in haemocytes following WSSV infection [4, 29]; and Xiao et al., 2025 in haemocytes under Decapod iridescent virus 1 (DIV1) infection [31].

While this pilot study is the first to provide a more detailed look at the different functional and supportive cell types found in Pacific whiteleg shrimp, future studies will be needed to round up these findings and help shed light into the different cell types that could not be captured in the present analysis. Larger studies processing and analysing more samples using the presented protocol will likely help increase the cluster prediction accuracy. Mapping the data onto newer genomes with better gene annotations will also help not only clarify the cluster identities, but also shed more light onto the expression profiles of control animals. Lastly, the protocol presented here will greatly benefit from further refinement and testing using different tissues in order to improve the nuclei recovery and minimise sample debris.

## Conclusion

This study developed and tested a new method for the isolation and processing of quality nuclei from frozen Pacific whiteleg shrimp hepatopancreatic tissue and employed single nuclei transcriptomic analysis to uncover the different cell types found in this important immune organ. While doing so, we also generated a list of gene markers specific to each cell type we encountered.

The sequencing data we generated was used to create one of the first cell atlases for Pacific whiteleg shrimp hepatopancreas, and the only one to investigate both hepatocyte and supporting cell clusters, including a separate group of intestinal epithelial cells which suggests a greater cellular complexity of the hepatopancreas. By combining the knowledge gained through this study with past and future work in other penaeid shrimp species, such as the more well-studied black tiger shrimp, we have the potential to create a powerful resource that will help uncover new knowledge about these important species, especially in the field of stress and immunity.

## Data Availability

The datasets analysed during the current study are available from NCBI BioProject database under BioProject Accession PRJNA1302321 which includes BioSample accessions SAMN50468451 and SAMN50468452. The BioProject is available online at: [https://www.ncbi.nlm.nih.gov/bioproject/PRJNA1302321/].

## Abbreviations

DEG: Differentially Expressed Genes
DIV1: Decapod iridescent virus 1
DNA: Deoxyribonucleic Acid
GEM: Gel Bead-In Emulsions
pH: Potential of Hydrogen
QC: Quality Control
RNA: Ribonucleic Acid
SCT: Single-cell RNA sequencing transform method (assay)
snRNA-seq: Single Nuclei RNA Sequencing
ST: Salt-Tris solution
TST: Salt-Tris solution with Tween 20
UMAP: Uniform Manifold Approximation and Projection
UMI: Unique Molecular Identifier
WSSV: White spot syndrome virus
